# Supplementary material for: Identification of QTLs for Spot Blotch Resistance in Two Bi-Parental Mapping Populations of Wheat
Source: Plants (Basel). 2021 May 13;10(5):973. doi: 10.3390/plants10050973 (PMC8153151; doi:10.3390/plants10050973)
Supplement: Supplementary file 1 [file plants-10-00973-s001.zip › plants-1189160-supplementary.pdf]

Table S1. Mean SB scores for parents, checks and RILs during 2013 – 2015 for WC and KC population.

		SB Scores			
**WC population**					
**Particulars**	**Parent/Checks/RIL**	**2013**	**2014**	**2015**	**Average**
Parents	WAXWING*2/CIRCUS	30.25	418.67	95.68	181.53
	CIANO T 79	43.21	532.41	126.85	234.16
Checks	CHIRYA.3	29.63	398.61	90.50	172.91
	FRANCOLIN #1	38.89	409.41	118.10	188.80
	SONALIKA	46.30	748.61	143.70	312.87
RIL	Susceptible (Max)	49.38	768.52	156.79	324.90
	Resistant (Max)	24.07	329.94	71.60	141.87
KC population					
Parents	WHEATEAR/KRONSTA	43.21	538.27	142.90	241.46
	CIANO T 79	43.21	811.73	150.93	335.29
Checks	CHIRYA.3	25.93	361.73	98.15	161.94
	FRANCOLIN #1	38.89	491.67	107.25	212.60
	SONALIKA	47.84	902.93	141.67	364.15
RIL	Susceptible (Max)	49.38	873.46	185.19	369.34
	Resistant (Max)	25.93	316.05	84.88	142.28

Table S2: QTLs identified for days to heading (DH) and plant height (PH) in WC and KC RIL populations.

**Population**	**QTL**	**Position (cM)**	**LOD**	**Marker interval**	**Physical range [a] (Mb on CS)**	**Mean PVE [b]**	**Trait/gene associated [c]**
WC	1A	80.5 - 83.5	6.0	2254313 - 1375359	501.0 – 506.5	3.6	PH
	2A	50.0 - 54.5	3.3	1018775 - 4008145	41.2–42.2	1.9	PH
	4B	37.5 - 39.5	22.9	Rht-B1 - 3958247	18.8 – 37.6	16.6	PH/Rht-B1
	4D	0 - 2.5	5.6	BS00036421 - 993587	32.3 – 209.2	3.3	PH/Rht-D1
	5A	156.5 - 158.0	17.3	1106357 - Vrn-A1	587.4 – 588.4	24.8	DH/Vrn-A1
	6A	59.5 - 60.5	27.6	1064961 - 4910893	76.6 – 77.5	20.1	PH
	7A	72.5 - 76.5	3.5	100148995 - 1075735	28.1	2.0	PH
					**Total PVE (%)**	**72.3**	
KC	1D	20.5 – 31.0	3.0	991664 - 1019993	10.9 – 18.4	3.7	DH
	2B	44.0 – 46.5	10.8	4993668 - 3947227	65.1 – 68.6	12.9	DH
	4B	39.0 – 52.5	20.9	Rht-B1 - 3222467	18.8 – 483.8	27.5	PH/Rht-B1
	4D	0 - 5.5	7.7	BS00036421 - 1059032	32.3 – 336.8	7.9	PH/Rht-D1
	5A	126.0 – 132.5	12.2	987242 - Vrn-A1	570.5 – 588.4	15.1	DH/Vrn-A1
	5A	148.0 – 150.5	4.2	4910865 - 3064766	611.5 – 616.5	4.9	DH
	6A	68.5 - 70.5	3.0	987538 - 1091565	100.7 – 453.3	2.8	PH
					**Total PVE (%)**	**74.8**	

[a]The physical position of QTL projected on IWGSC Chinese Spring RefSeq ver. 1.0 reference genome is shown in Megabase pairs.

[b] PVE – Percentage of explained phenotypic variation for the QTL in the mean year.

[c]DH: Days to heading; PH: Plant height

Table S3: Disease resistance-related genes identified within the confidence intervals of the stable QTLs in the WUYA x CIANO T79 population.

QTL	Position (cM)	Physical range (Mb in CS)	Gene identifier	Length (bp)	Putative functions
1B	175.5 - 178.0	670.6 - 673.7	TraesCS1B01G460100.1	7,900	Disease resistance protein (NBS-LRR class) family
2A	90.4 - 98.0	73.5 - 101.0	TraesCS2A01G123500.1	3,345	Disease resistance protein RPM1
			TraesCS2A01G131700.1	2,443	Receptor-like kinase
			TraesCS2A01G160500LC.1	489	LRR receptor-like serine/threonine-protein kinase EFR
			TraesCS2A01G129800LC.1	441	Endonuclease/exonuclease/phosphatase family protein
			TraesCS2A01G130400LC.1	9,163	Pleiotropic drug resistance ABC transporter
2D	71.5 - 73.5	61.8 - 65.7	TraesCS2D01G111500.3	5,570	NBS-LRR disease resistance protein-like protein
			TraesCS2D01G113100.1	992	Universal stress protein
			TraesCS2D01G124000LC.1	1,017	Serine/threonine-protein kinase
4B	39.5 - 41.5	37.5 - 65.1	TraesCS4B01G056600LC.1	816	disease resistance protein (TIR-NBS class)
			TraesCS4B01G050200.1	6,297	Histidine kinase
			TraesCS4B01G053900.1	716	late embryogenesis abundant protein
			TraesCS4B01G054700.1	19,403	Pleiotropic drug resistance ABC transporter
			TraesCS4B01G055500.1	804	Ethylene-responsive transcription factor
			TraesCS4B01G062600.1	4,259	Cathepsin B-like cysteine protease
			TraesCS4B01G069200.1	15,230	Serine/threonine-protein kinase
			TraesCS4B01G069500.1	1,893	WRKY transcription factor
4B	57.5 - 60.5	558.6 - 572.1	TraesCS4B01G282600.1	2,690	Calmodulin-binding family protein
			TraesCS4B01G283300.1	3,982	Serine/threonine-protein phosphatase
			TraesCS4B01G468400LC.1	1,549	Calcium-binding endonuclease/exonuclease/phosphatase family
7B	8.5 - 12.5	18.1 - 18.1	TraesCS7B01G020500.1	6,246	NRT1/PTR family protein 2.2

Table S4: Disease resistance-related genes identified within the confidence intervals of the stable QTLs in the KATH x CIANO T79 population.

QTL	Position (cM)	Physical range (Mb in CS)	Gene identifier	Length (bp)	Putative functions
2A	48.1 - 48.6	7.9 - 8.0	TraesCS2A01G018400LC.1	528	NB-ARC domain containing protein
			TraesCS2A01G016800.1	4,637	NBS-LRR-like resistance protein
			TraesCS2A01G016900.1	1,925	NB-ARC domain-containing disease resistance protein
2B	13.7 - 22.0	24.1 - 27.2	TraesCS2B01G050500.1	5,294	Disease resistance protein (TIR-NBS-LRR class)
			TraesCS2B01G050700.2	8,090	Subtilisin-like protease
			TraesCS2B01G052400.1	8,709	NBS-LRR class disease resistance protein
			TraesCS2B01G055000.1	8,332	NBS-LRR disease resistance protein-like
4B	31.8 - 48.0	21.6 - 483.8	TraesCS4B01G054700.1	19,403	Pleiotropic drug resistance ABC transporter
			TraesCS4B01G096900LC.1	4,978	NAC domain-containing protein
			TraesCS4B01G092600.1	5,531	Disease resistance protein RPM1
			TraesCS4B01G120400.1	1,624	Non-specific serine/threonine protein kinase
4B	56.5 - 57.0	535.1 - 548.2	TraesCS4B01G443600LC.1	5,048	NAC domain-containing protein
			TraesCS4B01G450300LC.1	330	ABC-2 type transporter family protein
4D	0 - 30.5	18.8 - 32.3	TraesCS4D01G042400.1	7,665	Wall-associated receptor kinase 2
			TraesCS4D01G045300.1	1,528	Accelerated cell death 11
			TraesCS4D01G039000LC.1	303	Serine/threonine-protein kinase WNK (With No Lysine)-like protein

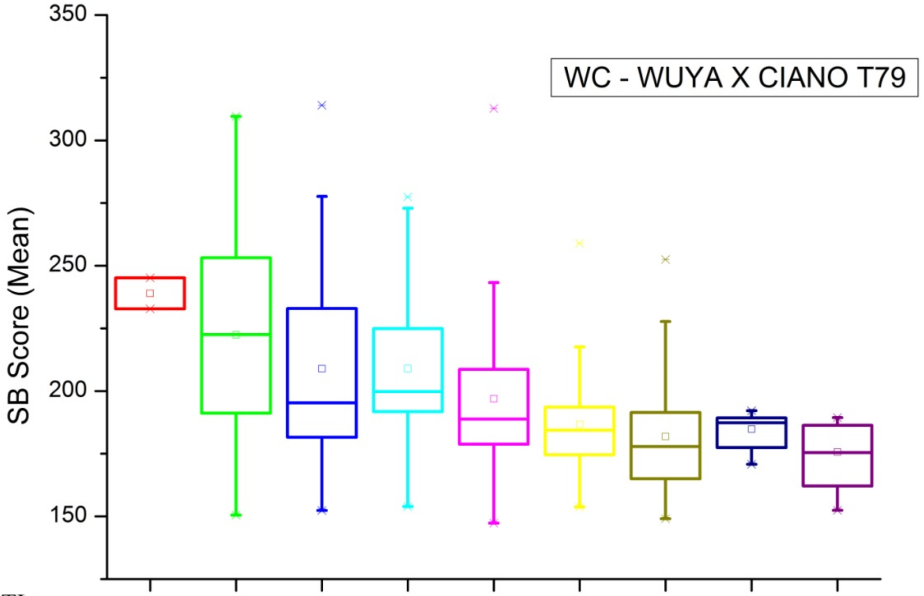

No. of QTLs	0	1	2	3	4	5	6	7	8
No. of RILs	2	19	43	39	35	43	31	8	10
SB Score (Mean)	239.0[a]	222.5[b]	208.9[c]	209.0[c]	197.0[d]	186.7[e]	181.9[f]	184.8[e]	175.7[g]

[a-g]Tukey's group indicated as superscript. Same letter indicates non-significant difference between the group.

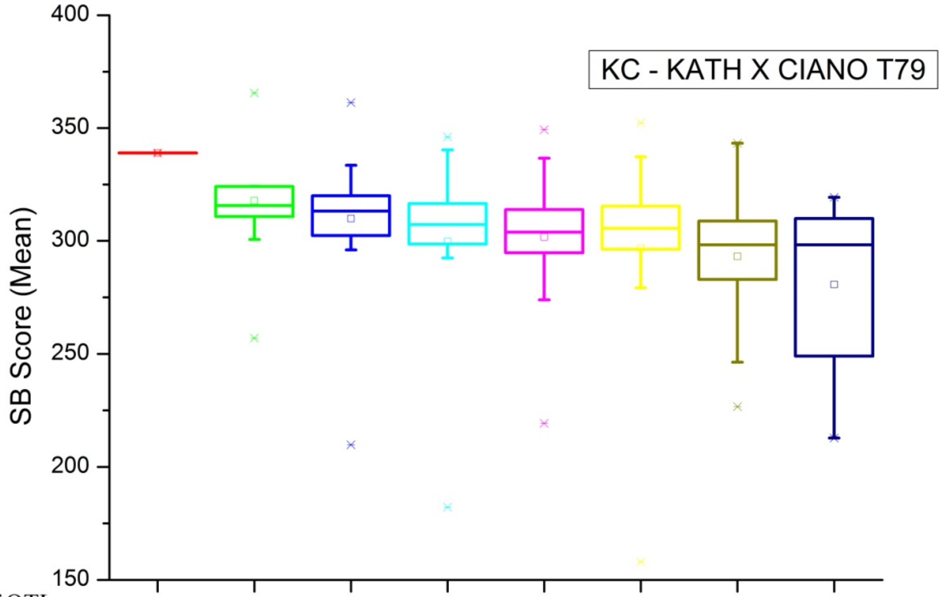

No. of QTLs	1	2	3	4	4	5	6	7
No. of RILs	1	13	24	51	54	46	28	13
SB Score (Mean)	339.0[a]	317.8[b]	309.9[c]	299.7[d]	301.8[d]	296.8[d]	293.2[e]	280.7[f]

[a-g]Tukey's group indicated as superscript. Same letter indicates non-significant difference between the group.

Figure S1. Effect of stacking multiple QTLs on spot blotch score in WC (WUYA X CIANO T79) and KC (KATH x CIANO T79) RIL populations.

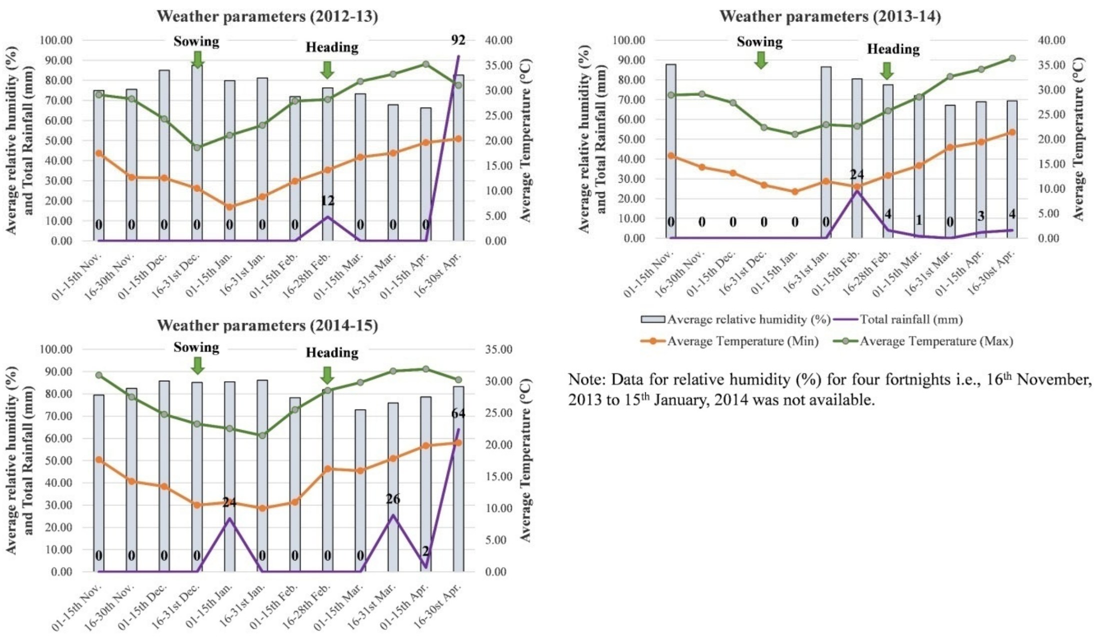

Figure S2. Weather parameters during the crop growth stages in three consecutive years (2013 - 15) from November to April.
